# Proceedings of the Regular Meeting of the Organon Society of Boston

**Published:** 1889-02

**Authors:** S. A. Kimball

**Affiliations:** Secretary


### PROCEEDINGS OF THE REGULAR MEETING OF ORGANON SOCIETY, BOSTON, MASS.

The first meeting of the Organon Society of Boston, Mass., since the adjournment last May was held at Dr. Bell's office, Thursday evening, December 20th. There were present Drs. Cobb, Bell, Davis, Dike, Dillingham, Dutton, Harvey, Hastings, Harris, Kennedy, L. H. Kimball, G. A. Kimball, Plummer, Stewart, Tompkins, Winn, and W. F. and W. P. Wesselhœft.

Dr. Bell read, beginning at Section 61.

Dr. Bell—Because some physicians have not "correctly observed and considered the deplorable results" of such treatment, we hear of such things as the following:

An old patient of mine, a lady seventy years of age, in an adjoining town, was accustomed to call in the physician there whenever she needed a little attention. He was supposed to be a homœopath, and generally did very well for her.

She complained so bitterly of constipation that he gave her some sugar-coated pills, which proved to be composed of Podophyllum. She would take one of these daily and have a natural stool each day when taking them; as a result, she was soon worse off than before. He was not a correct observer, or he would have known that the regularity of the stool would not continue long when produced by the Podophyllum.

Dr. Wesselhœft—Sections 64 and 65 illustrate the primary and secondary effects of drugs, and show the bad result of the anti-pathic method on account of the secondary effect. The secondary effect of Opium in large doses has often been observed to produce a diarrhœa, and in a case that has recently come under my notice, this was particularly marked.

Dr. Bell—We have all seen the secondary effects of the action of drugs, especially in patients coming from allopathic hands.

I think we can add to foot-note 63, the effects of anæsthesia and surgical shock, although Arnica is as valuable in surgical shock as brandy and that sort of thing.

Dr. Bell—In the first paragraph of Section 70 are we to include in our thought morbific influence?

Dr. Harris—Does it not refer to the pathology of the case which must not be considered, but we must treat the symptoms of the patient only?

Dr. Wesselhœft—I think Hahnemann means that we are to have no theories to prescribe for, nor should our minds be biased by any morbific or material working that would confuse us in the selection of the remedy, but we should depend on what we can see, hear, and feel. We should not theorize as to the cause of the disease. Of course, if we get a history of psora, syphilis, or a bad vaccination, that is to be considered, and enters into the symptomatology of the case.

Hahnemann was probably thinking of the theories that might come up in regard to disease.

Dr. Bell—We should not prescribe Mercurius for syphilis, or Sulphur for psora, unless they are indicated.

Dr. Kennedy—In the third part of Section 70, the words "According to experience" express a great deal, and seem to cover the ground. It is not from theories that Hahnemann draws these conclusions, but from actual experience.

Dr. Bell—According to some of our friends, experience is of no account, when we talk of cures made by high potencies. They say such experiences are of no value! In Section 73, where Hahnemann speaks of the second class of acute diseases, sporadic diseases, as being engendered by meteoric or telluric agencies, it is interesting to note that Dr. Lawson in the Milroy lectures, at the Royal College of Physicians of London, on Epidemic Influence, takes the same ground as Hahnemann concerning cosmic or telluric influence in the causation of epidemics. He says that the epidemic factors embrace large portions of the earth's surface at the same time, and that their course from year to year is somewhat definitely defined. Febrile epidemics pass uniformly to the northward, till they finally disappear. They occur periodically, every second year, or some multiple of two years, and like a series of waves pass over a greater or less portion of the earth's surface. The form of fever may be determined by local causes. He calls these influences "pandemic waves," and thinks they coincide with a greater dip of the magnetic needle.

Section 75 ought to modify our prognosis in cases that come to us after prolonged old-school treatment.

Dr. Wesselhœft—I can second that with all my heart, and I think that in many of our failures in chronic cases, which seem so simple when compared with others that have been cured, it will be found that such cases are almost always those that have come to us after being under palliative treatment for years and years. When a patient comes who has been taking Digitalis for a long time, little can be done. Something may be accomplished if the Digitalis has been left off for some time, but they usually come after a long-continued use of the drug. When we get a history of the abuse of Iodine, Digitalis, Mercurius, or Quinine, in continued dosing, in such cases we must make our prognosis very carefully.

Dr. Bell—I would add to the drugs just mentioned Salicylic acid. Homœopathic physicians are not alarmed by serious heart lesions, and if Digitalis has not been given, the patient will often live a long time in comparative ease.

I once had a patient who, before he came to me, was continually taking Iodide of Potassium or Colchicum for a rheumatic trouble; he had both of these drugs at home, and if he did not happen to feel just right before going down-town, he would take a dose of one or the other as it happened. But whenever there were signs of a rheumatic attack, then the dosing was *ad libitum*.

When he came under my observation, of course all that was stopped, and he became very much better in his general health; but, because I did not relieve him quite as quickly in one or two acute attacks as he thought his Colchicum did, he returned to it and his Iodide of Potassium. That was two years ago. I heard of him the other day; he is a complete wreck and has just returned from some European baths where he had gone in the vain pursuit of health.

He could not wait for the complete action of the homœopathic remedy, although he was getting better generally, but that was no trial of Homœopathy after years of such drugging.

Dr. Davis—We ought to tell such patients that the reason we cannot cure them is not because the case was incurable at first but because they were so drugged.

Dr. Wesselhœft—I do not think it is so much the effect of the drug itself as it is due to the reactive powers of the system being so weakened that they will not respond. My father, years ago, said he did not know what to do with cases of Iodine poisoning—that was when they used Iodine for everything. He got some good results by giving Iodine high at first. I recall a case of Digitalis poisoning that we had some time since; the patient came in a day or two ago, perfectly restored. It was a case of peculiar heart's action, and he had been dosed with Digitalis for some weeks. Dr. Bell saw him the second or third time he was visited, and because he had some marked Digitalis symptoms, particularly the great sinking at the stomach, he gave him Dig. in a high potency, and from that time he began to improve, and required no other medicine. The incurable Digitalis cases are found in organic heart-diseases, which have been fed on Digitalis for a long time. When we get cases after long-continued drugging, the same remedy in a high potency may help us out of a tight place.

Dr. Kennedy—May not that be so in cases of Rhus poisoning?

Dr. Wesselhœft—Yes.

Dr. Davis—Did not the patient in the case just mentioned recover because he had no heart-disease?

Dr. Wesselhœft—There was no organic lesion, but he had a very slow pulse, and was spitting a good deal of blood. He had no other remedy after the Digitalis, but it was repeated several times.

I wish now to show by the following case the results of repressive treatment, and what can be done by the indicated remedy in a few weeks after a suppression of five years' duration; it is not a case of drug influence but of suppression:

Miss ——, age forty-one, has had hemorrhoids as long as she can remember. Five years ago the external tumors were ligated. Before ligation they were very painful and tender, but did not bleed; she could not sit without great inconvenience. For two years after the operation she thought herself perfectly well.

The tumors gradually reappeared higher up in the rectum. For the last six months she has had profuse hemorrhages, coming on in spells lasting from two days to a week, with intermissions of never longer than a fortnight. With this there has also been prolapsus ani, even with moderate effort at stool, which reduces itself spontaneously.

The bleeding only occurs during stool; blood clear, bright-red, occasionally it spurts out so that it is heard to strike against the side of the vessel.

These attacks of bleeding are accompanied by great exhaustion. While the bleeding lasts she has no pain, but in the intervals the hemorrhoids swell and she has a dull, aching pain, which is again relieved by the bleeding. The aching is especially aggravated by walking, the stools are daily and rather soft; menses regular—painless. Has palpitation on ascending, is very anæmic, and has a profuse thick, yellow, non-irritating leucorrhœa. Her mother died thirteen years ago, at which time there was much sickness in the family, and she went through great anxieties and griefs. Her constitutional hemorrhoidal trouble has been much worse since then. Ignatia$^{cm}$ one dose, dry.

*A week later*—Stools harder, less bleeding, has still to rest after stool on account of dull ache, which lasts for hours. Walking aggravates the pain more than any other exercise. Sac. lac.

*A week later*—No bleeding, thinks prolapsus is better. Sac. lac.

*A week later*—No bleeding, scarcely any trouble with prolapsus, can walk after stool without aggravation. Palpitation much decreased, feels stronger, looks much better. Sac. lac.

*A week later*—No bleeding, no prolapsus, walks up-stairs with very little fatigue or palpitation, is not obliged to rest after stool, color in cheeks and lips, and is so much improved that her friends all remark it. One dose of Ignatia$^{cm}$ was all she received.

Dr. Kennedy—Do we not often attribute the causes of disease to operations when this is not so? I have a patient now who had his piles cauterized with the thermo-cautery two years ago. He now has locomotor ataxia. The trouble began about a year after the operation. Was that the cause of it?

Dr. Wesselhœft—I should take it as the cause. I have known removal of piles to cause intense congestion of the chest after a fortnight.

Dr. Bell—I recall two cases of rheumatic fever which, I think, were caused by excision of the tonsils. When we suppress a psoric thing we run a great risk.

Dr. Wesselhœft—If we remove the outward showing we have a metastasis of an expression. It is fortunate, very fortunate, if the expression returns in the same place as in the case I just read. Subjects with a bad family history are dangerous ones in which to suppress any manifestations.

Dr. Bell—A surgical line, however, must be drawn somewhere. In ovarian tumors it is often necessary to operate on account of the mechanical pressure.

Dr. Wesselhœft—Of course, surgery is a very necessary thing, but things that can be cured medicinally should not be suppressed surgically.

Adjourned to Thursday evening, December 27th.

---

### MEETING OF ORGANON SOCIETY, DECEMBER 27TH, 1888.

Dr. Wesselhœft being absent, Dr. Bell read, beginning at Section 75.

Dr. Bell—In regard to this paragraph, I think we hardly realize its truth, and do not impress it enough upon our patients.

I should like to have an expression from each of the members present on this subject to see what your experience has been with patients who have been drugged.

Dr. Dutton—It often seems to me to be due to a lack of recuperative power on the part of the patient, for while some will not respond, others, after having been severely dosed, will come out all right in response to the indicated remedy.

Dr. Bell—How is it with certain drugs; Quinine, for instance?

Dr. Dutton—I do not at present recall a case of Quinine dosing.

Dr. Cobb—I am more and more convinced that such is the case, that patients that have been drugged will not respond to homœopathic remedies; but there are some exceptions, and some times we do get a reponse.

Dr. Bell—Do you ascribe it to a lack of constitutional susceptibility or to the effects of drugs?

Dr. Cobb—Some cases will respond to a certain extent, although they cannot be made strong and vigorous, but others won't respond at all to well-selected remedies. These cases are always those that have been drugged abominably.

I am always discouraged with Quinine patients, but there is one that I recall who was drugged with Quinine for years. He had always been treated allopathically with Morphine and such drugs. After being under homœopathic treatment for some time, he now considers himself a well man. He is a young man, however.

Dr. Plummer—We seem to get quicker responses to our remedies with young children, and is it not for the reason that they have not been drugged? It seems to be more satisfactory to prescribe for young children.

Dr. Hastings—I recall a case of malaria which had been treated for some time with thirty grains of Quinine daily, but it was cured with Gelsemium$^{200}$. There was a complete cure in this case.

Dr. J. H. Payne—I remember a case of malaria under the influence of Quinine which yielded at once to a high potency of Ignatia, and permanently.

Dr. Davis—I have met a good many cases that have puzzled me, cases especially that had been in the army during the war. I did not know whether it was due to the drugging they received there or to their constitutions being affected by the exposure and hardships.

Dr. Jameson—I do not know that I have found patients that have been drugged particularly unresponsive. I recall a case in which a patient had a cancer removed from her breast about a year ago. Last summer she had pains in the region of the stomach, and vomited more or less daily for nine weeks. She took a good deal of Morphine and other drugs for the pain and vomiting. Six weeks ago I was called in, and gave her one dose of Hydrastis on account of great sinking at the stomach and other symptoms that I do not now recall. The first night was a good one, the second night she suffered considerably, but the vomiting stopped the third or fourth day and she has only vomited once or twice since. She responded very quickly to the remedy, and has been comfortable ever since.

Dr. Harvey—I have known cases that have been drugged—chronic cases—to respond to remedies for a time and then fail to respond. I recollect a case of cancer of the rectum that came to me after being under Morphine for a long time. I kept him comfortable for a time, and then, the pain getting worse, he returned to his Morphine physician, and after being under his care for a while he came back to me for relief, which was afforded him again for a time; he finally died under the care of the other physician.

Dr. Nichols—Patients who have taken homœopathic remedies indiscriminately are also hard to prescribe for, and we must select the remedy with great care after a good many remedies have been taken.

I have an interesting case now of cancer of the breast, which had been drugged for some time before coming under my care. There had been no cutting, and the patient has always been in good health; there is no history of grief or of a blow.

The most peculiar thing about it was the tubercular character of the growth—very hard, small nodules in the substance of the breast and a more superficial line of them to the axilla and out to the middle of the back. The only other important symptom was that she had had very severe headaches; since the cancerous growths appeared she has had no headaches. The nipple was retracted, and it had been pronounced cancer by the best allopathic authority here in the city. On account of the tubercular character of the growths and the former headaches, which were very similar to the ones that Dr. Swan says are an indication for Tuberculinum, I gave one dose of Tuberculinum. In forty-eight hours she began to have burning, lancinating pains in the growths. She had had some burning pain before, but the sedatives given had kept down most of the pain. The burning, lancinating pains lasted two or three days, then gradually disappeared. The tubercles have softened—becoming a little larger as they grow softer—the retracted nipple is not so much retracted; she is very nearly free from pain, and her general health is excellent. Of course, we do not know how it will come out, but the progress so far is very satisfactory, and the case is interesting, both on account of the diagnosis and the remedy given.

Dr. Bell—You do not always have pain in scirrhus. I have removed two cancers lately in which there had been no pain.

Dr. Nichols—I also recall a case of ascites, which had been allopathically abused, in which the relief was so quick from Apis the ascites disappearing as if it had been baled out, that Dr. Wesselhœft said at the time that he did not think the case would recover, the quick result being probably only a palliative effect. He was correct, the ascites gradually returned, and the case would not respond again.

Dr. Kennedy—When cases that have been drugged come to me, and where there is no remedy indicated, I give an antidote or Sac. lac., and wait for something to develop.

Dr. Bell—The general opinion seems to be that cases will respond even after prolonged drugging. I will now ask Dr. Kimball to read a translation by Dr. Wesselhœft from Bœnninghausen's "Aphorisms."

Dr. Kimball—"Those who without visible causes are subject to severe attacks of syncope usually die unexpectedly. (Hippocrates.)

"When we consider the low stage of anatomical knowledge at the time of Hippocrates, it is not surprising that he should have been ignorant of the probable cause of such fainting spells, which may be due to organic lesions of the heart or its large blood-vessels.

"A word of warning may not be out of place here against the use of heroic antipathic drugs for the frequently occurring palpitations, which use often induces such lesions as aneurisms and ossification, or largely promotes their development. We have observed this fact most frequently after the use of the favorite and popular Digitalis purpurea, which in these days is given for *every* palpitation of the heart, in excessive doses, and is so deceitful and seductive on account of its antipathic primary action. It may not be superfluous to repeat our advice given twenty-seven years ago to the younger homœopaths, viz.: to be cautious about accepting such patients who are now for the first time ready 'to make a trial of Homœopathy.' It will be wiser to refer them back to their former physicians. Nothing can be gained in the way of reputation, or reverence for Homœopathy with this class of cases, as the most carefully selected remedies prove impotent, and the usually sudden death is charged to Homœopathy."

Dr. Hastings—What is meant by "invented" in Section 70?

Dr. Bell—The original is "thought out"—that is, a distinction is to be made in the selecting of drugs to be proved.

Dr. Cobb—Speaking of the effects of drugging, I now recall a case of a man who is a hopeless wreck from the effects of overdrugging; he may live, but he can never be cured. Years ago he was mercurialized, then he went through a course of Thompsonian treatment, then he took sulphur baths, and then he was treated by electricity and the needle would be passed into him to the depth of three inches to relieve him of pain. He has now a constant pain in one spot that troubles him a good deal. Last year he had a severe attack of sciatica, and he would sometimes respond almost immediately to well-selected remedies, but the effect would not last long, the pain would soon return, and the attack seemed to wear away of itself; the remedies did not cure him.

Dr. Bell—Since Hahnemann's time they have more weapons of danger, such as strophanus, convallaria majalis, antipyrin, antifebrine, etc. A physician told me recently of a case of typhoid fever that had been treated with antipyrin, in which an arm and leg had to be amputated on account of gangrene. He thought the gangrene was due to the antipyrin; he is quite a doser himself, and if he thought so, I may think it might have been due to the same cause.

Dr. Davis—In regard to Section 77, rest will certainly relieve an overtaxed mind.

Dr. Bell—We often have cases from grief or an overtaxed mind, and we must tell them that medicine will not take the place of rest; they must get that, and then the remedy will relieve them much more quickly. Rest is especially needed in cases of continued worries and griefs.

Dr. Payne—Medicine will certainly relieve cases of grief.

Dr. Hastings—I have a case of a mother who lost her daughter four months ago, and her grief is as fresh now as ever in spite of remedies.

Dr. Bell—I have now the case of an old man who has been going to business regularly, but only for a short time daily, because he liked it. I had told him he must not go, but as it was a pleasure to him to go and sign a few checks, he thought it could do him no harm. The other night he had an attack of aphasia, and now, of course, he must have absolute rest.

Dr. Davis—Does Hahnemann mean that we are not to give a remedy?

Dr. Bell—Certainly not, but rest or change must be insisted on. In regard to the disease mentioned in Section 80, as arising from psora, perhaps nervous debility might be questioned as always being caused by psora. I think it is, however. I have a case of nervous debility in a very healthy looking woman of about forty years of age, and it is all traced to a bad vaccination in childhood, where the matter was taken from the arm of another child. That child was undoubtedly psoric. Psora does not always retard development. We cannot always explain these things satisfactorily to patients, but as blood-poisoning is quite a common expression now, we can tell them that these things are due to deeply-acting blood-poisonings, and that is always very satisfactory to them.

Adjourned to January 10th, 1889.

S. A. Kimball, *Secretary.*

